# Equine Headshaking Syndrome: Triggers, Seasonality, and Treatment Efficacy in Australia

**DOI:** 10.3390/ani14060875

**Published:** 2024-03-13

**Authors:** Teagan Bell, Panoraia Kyriazopoulou, Camilla Mowbray, Barbara A. Murphy

**Affiliations:** 1 School of Agriculture and Food Science, University College Dublin, Belfield, D04 V1W8 Dublin, Ireland; 2 Equilume Ltd., Naas, W91 TP22 Co. Kildare, Ireland; 3 Independent Researcher, Galston, NSW 2159, Australia

**Keywords:** horse, headshaking, triggers, symptoms, treatments, seasonality, trigeminal-mediated headshaking syndrome, Australia

## Abstract

**Simple Summary:**

Equine headshaking syndrome is a painful condition causing the horse to shake or flick its head violently without any obvious reason. Treatments for the condition are poorly effective and information on the condition in Australian horses is lacking. An online survey was sent to owners of headshaking horses in Australia to gather more information on when headshaking occurs and what treatments have been used to address it. The results showed that more geldings were affected than mares, and geldings were older when they first started headshaking compared to mares. Bright sunlight, wind, and high pollen count were the most reported triggers, and more than half of the owners reported that headshaking occurred in a specific season each year. Most owners had used more than two types of treatment, but few treatments were reported effective when used alone. The finding that a large proportion of horses start headshaking in spring and summer, combined with owner reports that light-blocking masks do not work well as a treatment, suggests that day length rather than brightness should be considered as an important factor in headshaking. This study provides new information on the causes and treatments of headshaking in horses in Australia.

**Abstract:**

Equine headshaking syndrome is a poorly understood neuropathic pain condition presenting as uncontrollable shaking, flicking, or striking of the head. Therapeutic options are limited, and treatments are only partially successful. Currently, epidemiological information on headshaking in the Southern Hemisphere is lacking. An online survey was circulated to Australian owners of headshaking horses to collect information on triggers, symptoms, seasonality, treatments, and perceived treatment efficacy. The responses (n = 216) showed the mean age at symptom onset as 9.6 (±4.7) years. More geldings were affected than mares (76% vs. 24%), and symptom onset occurred later in geldings compared to mares (10.1 ± 4.7 vs. 7.9 ± 4.0 years; *p* < 0.01). Bright sunlight, wind, and high pollen were the most commonly reported triggers (61%, 46% and 40%, respectively), and seasonal onset of symptoms was reported by 54% of respondents. In total, 71% of respondents reported using two or more treatments. The most common treatments were supplements (68%), nose nets (63%), light-blocking masks (48%), bodywork (48%) and pharmaceutical compounds (38%). Overall, treatments were considered ineffective by 33% of respondents. The findings were in agreement with surveys from the Northern Hemisphere. Of note was the perception of bright light as a primary trigger, alongside the reported low treatment efficacy of light-blocking masks. Seasonal intensification of symptoms and its relationship to day length merits further exploration.

## 1. Introduction

Headshaking Syndrome (HS) is a poorly understood condition in horses that reportedly affects 4.5% of the UK equine population and 1% of all equines [1,2]. It is characterised by spontaneous, involuntary, violent flicking of the head, snorting, rubbing the face on legs or objects and striking at the nose with a forelimb [3,4]. Symptoms tend to worsen under specific environmental conditions or when horses are exercised [3,5]. Affected horses may exhibit mild to severe symptoms that impact welfare and horse–owner interactions [2,3].

HS affects adult horses of all breeds, but geldings tend to be affected more often than mares in owner surveys [2,4]. Seasonal symptoms occur in approximately 60% of cases, with onset in spring or summer and cessation of symptoms during the winter months [2,3,6], indicating the presence of a photic-related component to headshaking [6].

Headshaking behaviours can occur due to many causes. Some uncommon ones include dental issues, tumours, ear mites, inflammation of the middle ear structures, guttural pouch diseases, problems arising from ill-fitted tack or upper airway and nasal problems, all of which can be addressed and treated appropriately once identified [7,8,9]. However, in cases where there is no apparent cause, idiopathic headshaking syndrome is usually diagnosed. This is now more commonly referred to as trigeminal-mediated headshaking syndrome (TMHS) since it was demonstrated that hyperexcitability of the trigeminal nerve is associated with the behaviour [10]. Despite this, the pathophysiology of the condition is still unclear.

HS has an extensive range of symptoms and triggering factors. These include long bright days, exercise, and weather such as rain, wind, and heat [2,9]. To date, very few scientifically controlled trials have been conducted to evaluate the effectiveness of potential HS treatments. Therefore, there is no single recommended treatment, such that therapeutic options for HS are limited, and treatments used to help manage the condition vary widely and are often only partially successful [4,11]. Treatments most used include pharmaceutical compounds, supplements, bodywork, light-blocking masks, nose nets and surgical interventions [4,9,12,13]. Pharmaceutical treatment options, such as the psychoactive medications carbamazepine and cyproheptadine, have shown promise in the remission of clinical signs in some horses, but require life-long therapy [9]. Sodium cromoglycate eye drops have also been shown to offer some resolution in horses with seasonal onset HS [14]. Dexamethasone use, which is widespread for the treatment of asthma [15,16], has been reported as ineffective for treating HS [2,17]. 

Surgical management of HS includes infraorbital neurectomy [18,19], chemical sclerosis [9] and cryotherapy of the infraorbital nerve [20,21], all with poor to moderate reported effectiveness. Percutaneous electrical nerve stimulation (PENS) has shown success as a treatment, with 86% of horses achieving remission, but repeated therapies at multiple intervals have been required to maintain this remission [2,21]. 

Apart from two studies that demonstrated positive effects of magnesium on headshaking behaviours [22,23], there have been very few controlled clinical trials evaluating supplement efficacy for HS [24]. However, owner perceptions support the findings that magnesium with salt supplementation may reduce symptoms [12]. 

Nose nets have been reported to have a transient positive effect [18,25] but this can depend on the specific symptomatic headshaking behaviour exhibited (e.g., vertical shaking vs. rubbing behaviours) [13]. Face masks used as a treatment for HS can be meshed, or light-blocking with UV or darkened shades [4,8,9,18]. Generally, success rates with face masks range from 2% to >50% [18,23]. Madigan et al. (1995) observed variable remission of headshaking when conducting a clinical examination under dark conditions or when using dark lenses in four horses [6]. However, a later study reported that the use of UV eye shades did not improve headshaking symptoms [18]. Homoeopathy and alternative therapies such as acupuncture and chiropractic manipulation have demonstrated minimal effects on HS [1,2,12].

Despite several studies exploring headshaking epidemiology, prevalence, symptoms and treatments in the UK, USA, and Europe [1,2,4,18,26,27], minimal epidemiological data exists for horses with HS in the Southern Hemisphere. For this reason, the present study aimed to evaluate owner responses to questions on HS symptomatology and seasonality, determine the type of treatments being used, and explore their reported effectiveness in Australia.

## 2. Materials and Methods

### 2.1. Survey Design

An online survey (see Supplementary Materials) comprising 18 questions was designed using SurveyMonkey and distributed publicly to Australian equine interest groups on Facebook. The survey consisted of questions with multiple-choice answer selections related to headshaking horse ownership, veterinary diagnosis of HS, information on horse age, sex, symptoms, age at symptom onset, symptom seasonality, and symptom triggers. Trigger options presented included bright sunlight, artificial light, cool breeze, wind, light rain, heavy rain, snow, dust, dark places, and high pollen count. Respondents had the option to add comments outlining additional triggers in an open comment box. Survey questions also aimed to collect information on horses’ lifestyle, the perceived impact of symptoms on horses’ welfare and respondents’ relationship with their horse. Further questions related to treatments used by owners to manage HS symptoms. Treatments were categorised as pharmaceutical, surgical, supplements, bodywork, nose nets and light-blocking masks. Respondents could multi-select from these options and add other treatments to an open comment box. 

Respondents were asked to indicate whether the treatments were effective by selecting ‘yes’ or ‘no’. If they selected ‘yes’, the respondents were directed to provide additional comments outlining what treatment was effective and describe the extent to which it was effective.

### 2.2. Data Analyses

The data were drawn directly from the online survey software and checked for inconsistencies in responses. Descriptive statistics explored relative frequencies (%) for all data. Non-parametric Mann–Whitney tests were conducted to assess differences between geldings and mares concerning age, age at symptom onset and number of years horses had been experiencing headshaking symptoms. Statistical analyses were carried out on GraphPad Prism (Version 10.0.2, 2023) and Microsoft Excel (Microsoft^®^ Excel^®^ for Microsoft 365, Version 2309, 2023).

## 3. Results

The survey resulted in 376 responses from multiple countries. Of those, 216 completed surveys were collected from owners of HS horses in Australia and used for further analyses. The number of responses to different parameters varied due to instances of non-response to specific questions.

### 3.1. Description of Respondent-Owned Horses

Geldings, mares, and stallions represented 75.9% (n = 164), 23.6% (n = 51) and <1% (n = 1) of the respondent-owned horses, respectively. The overall mean age reported by 214 respondents was 13.1 years (±5.8). Horses’ age at headshaking symptom onset ranged from birth to 30 years and was reported by 201 respondents. Mean age at onset differed between mares and geldings (10.0 ±4.7 vs. 7.9 ±4.0; *p* = 0.0057). At the time of taking the survey, the mean number of years respondents (n = 197) reported their horse experiencing HS symptoms was 3.6 (±4.1) years (Table 1). Finally, 50.9% (n = 110) of respondents reported having received a HS diagnosis for their horse from a veterinarian.

Half of the respondents’ horses (48.6%; n = 105) were used as ridden horses for competition; 31.9% (n = 69) were used as ridden horses for trails and recreation; 8.8% (n = 19) were part of a herd; 4.6% (n = 10) were ridden, working horses; 2.8% (n = 6) were companions to humans; 2.3% (n = 5) were used for groundwork/liberty only, and 0.9% (n = 2) were ridden, high-performance horses. The severity of HS symptoms reportedly led 7.4% of survey respondents (n = 16) to retire their horse and stop ridden work.

Concerning the perceived impact of HS on horses’ quality of life, 31.4% (n = 68) of respondents reported that HS impacted their horse ‘significantly’, 43.5% (n = 94) reported that HS affected their horse ‘somewhat’, and 25.0% (n = 54) reported that HS impacted their horse ‘minimally’. Almost half of the respondents (48.6%; n = 105) reported that HS symptoms had ‘significantly’ affected their relationship with their horse, 28.2% (n = 61) reported it affected it ‘somewhat’, and 23.1% (n = 50) were ‘minimally’ affected.

### 3.2. Triggers and Symptoms

**Figure 1:**
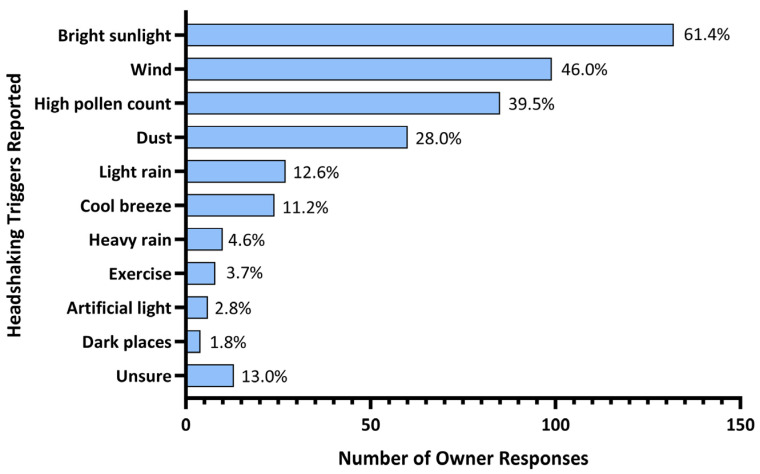
Triggers for headshaking syndrome reported by Australian horse owners.

One respondent did not report any triggers and was not considered in the subsequent analyses. Bright sunlight was considered a headshaking trigger by 61.4% (n = 132) of respondents and 14.8% (n = 32) reported it as the only trigger for their horse. Additional triggers reported were wind (46.0%; n = 99), high pollen count (39.5%; n = 85), dust (28.0%; n = 60), light rain (12.6%; n = 27), presence of a cool breeze (11.2%; n = 24), heavy rain, (4.6%; n = 10), exercise (3.7%; n = 8), artificial light (2.8%; n = 6) and dark places (1.8%; n = 4). Triggers were not obvious, or difficulty identifying triggers was reported by 13.0% (n = 28) (Figure 1).

Further triggers reported in the open comment field for this survey question included consumption of a high grass diet (n = 9), lucerne hay (n = 3), or clover hay (n = 2); hot weather or heat (n = 4); flies or insects on the face (n = 8); bathing or washing (n = 3); having tack/bridles/head collars on (n = 2); changes in light (n = 3); high humidity (n = 3); injury or previous surgery (n = 2).

The most commonly reported HS symptoms were ‘tossing head up and down’ (79.6%), ‘rubbing face on legs’ (66.2%) and ‘snorting regularly and/or sneezing’ (59.2%). A summary of all reported symptoms is presented in Table 2. 

### 3.3. Seasonality of Symptoms

**Figure 2:**
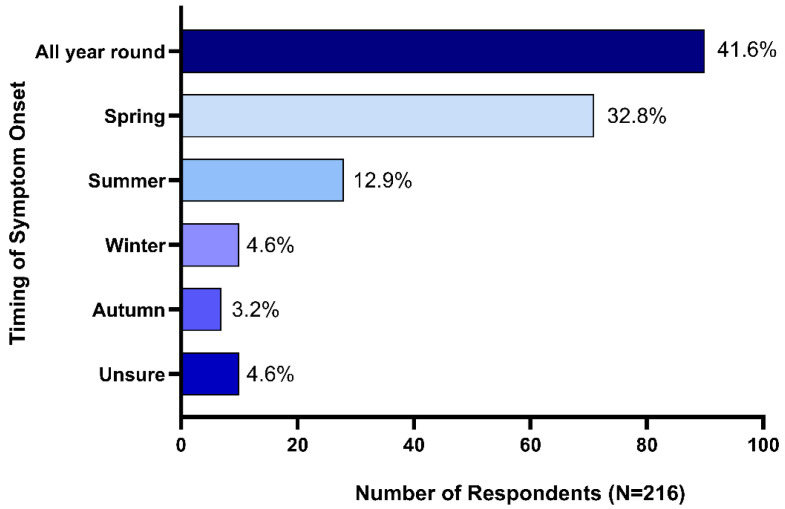
Seasonality of headshaking syndrome symptom onset.

Seasonal onset of symptoms was reported by 53.7% (n = 116) of respondents, 41.6% (n = 90) reported that their horses exhibited HS symptoms all year round, while 4.6% (n = 10) reported that they were not sure if HS symptom onset was impacted by season. Spring onset was reported by 32.8% (n = 71) of respondents, summer onset by 12.9% (n = 28), winter onset by 4.6% (n = 10) and 3.2% (n = 7) reported autumn onset (Figure 2). 

### 3.4. Treatment Options

**Figure 3:**
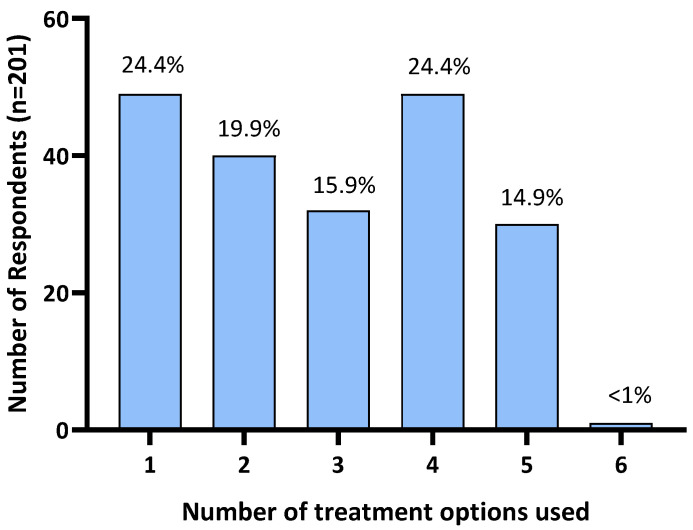
Number of treatments used by Australian survey respondents to manage their horse’s headshaking symptoms.

Of the 216 respondents, 15 (6.9%) reported not using any treatments. Further analysis was restricted to the remaining 201 respondents who reported treating their horse for HS. A quarter of respondents (24.4%; n = 49) reported the use of a single treatment. The remaining 75.6% reported using two or more treatments: 19.9% (n = 40) used two treatments, 15.9% (n = 32) used three treatments, 24.4% (n = 49) used four treatments, 14.9% (n = 30) used five treatments, and one (<1%) used six treatments (all treatment options provided in the survey; Figure 3).

**Figure 4:**
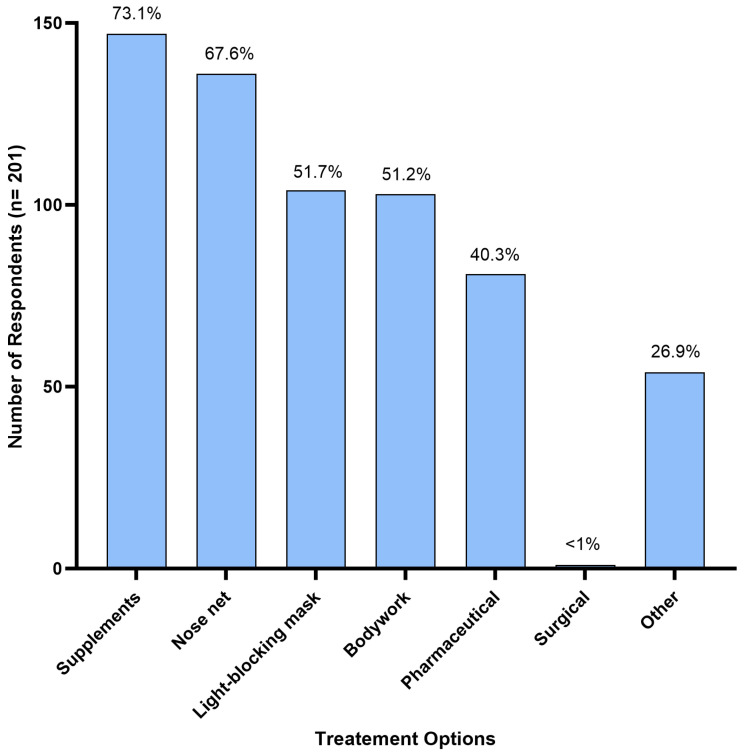
Treatment options used by Australian respondents (n = 201) to manage their horse’s headshaking symptoms.

Supplements were the most reported treatment option, used by 73.1% (n = 147) of respondents. Nose nets were the second most used treatment, used by 67.6% (n = 136) of respondents, and light-blocking masks were used by 51.7% (n = 104) of respondents to manage HS symptoms. Bodywork was used by 51.2% (n = 103) of respondents, followed by pharmaceuticals at 40.3% (n = 81); and <1% (n = 1) reported their horse having undergone surgical treatment (Figure 4). Additionally, 26.9% (n = 54) of respondents reported using various alternative treatments not mentioned in the survey. These included homoeopathic remedies (n = 1), acupuncture (n = 6), craniosacral therapy (n = 1), electrotherapy/electro-neural stimulation (n = 5), cortisone injections (n = 1), diet modifications (restriction of grass intake, removal of grain/high sugar diets, low potassium diets) (n = 12), ear nets (n = 2), fly masks (n = 4), cooling the horse down (using fans and misters, stabling on a hot day) (n = 3), and an Equilume^TM^ blue light mask (n = 1). Six horse owners reported complete resolution of HS symptoms following use of a mycotoxin binder, changing supplements to adjust for high blood potassium levels, a light blocking mask, craniosacral therapy, combined acupuncture and chiropractor treatments, and an Equilume^TM^ blue light mask, respectively.

### 3.5. Perceived Effectiveness of Treatments 

To facilitate the interpretation of reported effectiveness, we adopted the following categories to determine the effectiveness of individual treatment options: (1) effective: when a single treatment was used and reported to reduce HS symptoms; (2) potentially effective: where a reduction in HS symptoms was reported when a treatment was used in combination with one or more other treatments (i.e., if three treatments were used, each of those treatments was categorized as ‘potentially effective’ for that respondent); (3) not effective: where a treatment or combination of treatments were considered ineffective at reducing headshaking symptoms (i.e., if three treatments were used, all three were categorized as ‘not effective’ for that respondent).

**Figure 5:**
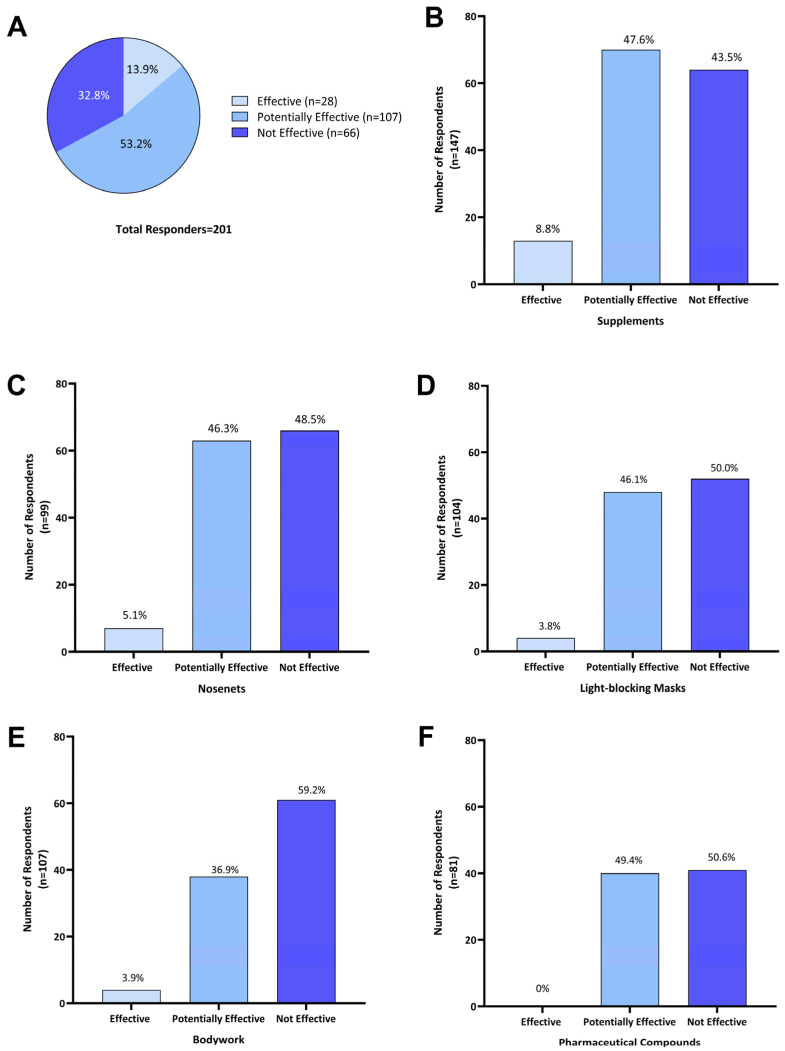
Perceived effectiveness of treatments as reported by Australian owners of headshaking horses for (**A**) all treatments (n = 201); (**B**) supplements (n = 147); (**C**) nose-nets (n = 136); (**D**) light-blocking masks (n = 104); (**E**) bodywork (n = 103); and (**F**) pharmaceutical compounds (n = 81). Effectiveness of individual treatment options was categorized as the following: ‘Effective’—when a single treatment was used and reported to reduce HS symptoms; ‘Potentially effective’—when that treatment was used in combination with one or more other treatments and a reduction in HS symptoms was reported; and ‘Not effective’—where a treatment was used either on its own or in combination with other treatments and no reduction in headshaking symptoms was reported.

Of the 201 respondents who treated their horse for HS symptoms, 67.1% (n = 135) reported that treatments were effective to some extent at reducing HS symptoms. As 53.2% (n = 107) used two or more treatments concurrently, these were categorized as ‘potentially effective’. Single treatments were reported as ‘effective’ by 13.9% (n = 28) of responders, while 32.8% (n = 66) of respondents reported that all treatments explored had no effect (Figure 5A). 

Of the 201 respondents using one or more treatment options, 73.1% (n = 147) reported using supplements. Of those, 8.8% (n = 13) reported that supplements were effective in reducing HS symptoms, 47.6% (n = 70) reported potential effectiveness when in combination with other treatments, and 43.5% (n = 64) did not consider supplements effective (Figure 5B). Supplements used by Australian survey respondents included electrolyte patches, training/calming supplements (magnesium, vitamin B and tryptophan), equine gut supplements, mycotoxin binder, chelated calcium, and macrobiotic salt.

Of 136 respondents who used nose nets, 5.1% (n = 7) reported that they were ‘effective’ as a sole treatment, while for 46.3% (n = 63) they were ‘potentially effective’, and 48.5% (n = 66) found them ‘not effective’ (Figure 5C). Fifteen owners who reported using nose nets in combination with supplements commented that this reduced HS symptoms during exercise.

Light-blocking masks were used by 51.7% (n = 104) of respondents; 3.8% (n = 4) reported their use as ‘effective’ when used as a single treatment; for 46.1% (n = 48) they were ‘potentially effective’ when combined with other treatments; and 50.0% (n = 52) found them ‘not effective’ (Figure 5D).

Of the 51.2% (n = 103) of respondents who reported using bodywork as a treatment, 3.9% (n = 4) considered it ‘effective’ in managing HS symptoms, for 36.9% (n = 38), it was ‘potentially effective’ when combined with one or more treatments, and 59.2% (n = 61) indicated it was ‘not effective’ (Figure 5E). 

Pharmaceutical compounds were used by 40.3% (n = 81) of Australian survey respondents and these included dexamethasone, cortisone, antihistamines, cyproheptadine, ivermectin, phenylbutazone, sucralfate and progestogen. When used in combination with other treatments, they were ‘potentially effective’ for 49.4% (n = 40) of respondents, while 50.6% (n = 41) reported that using pharmaceuticals was ‘not effective’ in managing HS symptoms (Figure 5F). More specifically, four owners reported the use of steroids (dexamethasone and cortisone) having a transient effect. Two respondents felt that cyproheptadine reduced symptom severity but did not lead to complete symptom resolution, while two other respondents reported some improvement with other undisclosed antihistaminic compounds. 

Only one respondent reported the use of surgical treatment and reported positive results. As it was combined with additional treatments, it could only be classed as ‘potentially effective’. No further information on the type of surgical treatment used was provided.

## 4. Discussion

The present study is the first to assess the signalment, symptomology, seasonality, treatment and perceived treatment efficacy of equine headshaking syndrome (HS) in Australia, a behaviour presentation associated with the absence of any apparent cause. Previous surveys of owners of HS horses have been conducted in Europe and Northern America [1,2,4,18,26,27]. In agreement with previous studies [1,2,4,8,27,28,29], the syndrome was over-represented in geldings (75.9%). It was proposed that geldings may be predisposed to trigeminally mediated headshaking syndrome (TMHS) due to the absence of normal negative feedback on springtime gonadotropin-releasing hormone (GnRH) release via testicular testosterone, leading to instability of the trigeminal nerve and the development of neuropathic pain [30]. In support of this, reproductive hormones have recently been shown to play a role in pain mediation in humans [31], and more specifically in human patients with trigeminal neuralgia [32]. While higher seasonal concentrations of gonadotropic hormones have been reported in geldings compared to stallions [33,34], concentrations of luteinizing hormone between HS and healthy geldings have not been shown to differ [28]. Nevertheless, the seasonality observed in the onset of clinical signs and the higher prevalence in geldings suggest some hormonal involvement.

Mean age at HS symptom onset (9.6 years) reflected previous findings, where onset ranged from 8 to 12 years [4,9,27]. However, in this study, symptoms were reported to occur two and a half years earlier in mares compared to geldings. There have been no previous reports of a difference in age at initiation of HS symptoms, so this is a novel finding. The age at which male horses in this study were castrated was not ascertained. It is possible that castration that occurred later in life, following sexual maturity, may have delayed the onset of this condition, whereby sufficient negative feedback by testosterone on gonadotropins occurred for a number of seasons, preventing the hypothesized instability of the trigeminal nerve [30]. 

Half of the respondents in this study confirmed they had received a veterinary diagnosis of HS for their horses. For the remainder, the proportion of horses truly exhibiting TMHS cannot be determined. Diagnosis can be a long and arduous process of elimination and currently the proposed method of excluding secondary causes of headshaking is a computed tomography scan, a costly and often impractical test [11,35]. In a review of 100 clinical HS cases, Lane and Mair (1987) reported that for 90%, a cause could not be determined, and a diagnosis of idiopathic headshaking was assigned [29]. With the more recent finding of involvement of the trigeminal nerve [36], the term ‘idiopathic’ has been superseded by ‘trigeminally mediated’ [2,18]. Therefore, it can be assumed that the majority of the respondents’ horses that were undiagnosed were likely exhibiting symptoms of TMHS. This is supported by the efforts of owners to relieve their horses’ symptoms, as evidenced by the numerous and varied treatments that were being used. Thus, despite unconfirmed diagnoses, the information collated from this survey provides relevant new information on signalment and therapeutic interventions for HS in Australia.

The majority of respondents (88.4%) owned ridden horses (competition, trails and recreation, working, high performance). According to 48.6% of owners, HS symptoms had significantly impacted the horses’ quality of life or the owners’ enjoyment of the time spent with their horse (31.4%). Additionally, sixteen owners reported that they had to retire their horse from a more active lifestyle specifically as a consequence of HS symptoms. Similar numbers were reported to have retired their horses due to HS in a survey of owners in France and Switzerland [27]. 

Generally, TMHS is considered a seasonal condition with symptom onset most frequently occurring in spring and summer [1,2]. In the present survey, over half of all respondents reported seasonal onset of symptoms, the majority in spring and summer. However, a large proportion (41.6%) also reported that their horses showed HS symptoms all year round. Of these, it was not determined whether intensity of symptoms may have varied over time of year. Roberts (2019) previously highlighted that only approximately 25% of horses with HS are symptom-free at a particular time of year [2]. This difference in the reported proportion of horses displaying seasonality of symptoms between studies may be related to different interpretations of the term ‘seasonal onset’ in survey questions that require a ‘yes’ or ‘no’ response. The nature of these questions potentially fails to capture perceived changes in symptom intensity across seasons. We suggest that defining a seasonal component for HS should encompass horses that transition from complete absence of symptoms during some months to recurring symptomology at specific times of year, and horses that display symptoms all year round, but where clear intensification of symptoms occurs in specific seasons. In this way, the proportion of seasonal headshakers could be better quantified and may allow us to focus on the pathophysiological causes.

Irrespective of whether seasonally or non-seasonally affected, reported symptomology in horses varies considerably depending on weather and environmental conditions. Sunlight was the highest reported environmental trigger in the present study (61.4%). This finding is in agreement with Ross et al. (2018) [1] who reported that 79.4% of horses were triggered by sunlight and Madigan and Bell (2001) [4] who reported that 52% of HS horses were affected by bright sunny days. Evidence connecting photophobia with trigeminal nerve activity has been demonstrated in humans and rats [37,38], and a photic-induced neuralgia pathway involving the trigeminal nerve was previously proposed in HS horses [6].

Supplements were the most frequently reported treatment for HS symptoms by Australian owners (73.1%). However, their use in combination with other treatments renders assessment of efficacy difficult. Nevertheless, the use of magnesium and mineral salts was most often mentioned. There is a known link between magnesium and alleviation of neuropathic pain in humans [39,40,41]. It is thought to occur by reducing catecholamine release and blocking neuromuscular calcium channels [22,42]. In horses, the addition of magnesium and boron to the diet led to a significant reduction in headshaking, with the most severely affected horses exhibiting the greatest benefit [23]. An imbalance in the magnesium/potassium ratio also correlates with the reports from some survey respondents that high potassium diets triggered headshaking symptoms. Magnesium regulates potassium transport intracellularly via multiple channels [41]. While the clinical evidence of effectiveness is still limited, magnesium supplements appear to offer a low-cost and widely available treatment option for HS.

Use of nose nets was deemed potentially effective in 46.3% of cases. However, only 5.1% of respondents considered them effective when used as the sole treatment. Nose nets may serve to reduce aversive stimulation of hypersensitised facial areas or act as an alternative sensory stimulus to distract from pain [12]. An owner perception survey in the UK involving more than 100 horses found that >25% reported some relief from wearing nose nets, despite not fully resolving the symptoms for all. Newton et al. (2000) reported that the use of occlusive nasal masks improved symptoms in more than half of the twenty horses examined [9]; however, this treatment option was not used by survey respondents in Australia.

Despite sunlight representing the primary reported trigger of HS by Australian owners, only 3.8% of respondents considered light-blocking masks effective when used alone, while 50% found them ineffective. Reducing exposure to sunlight using blindfolds, light-blocking masks and dark stalls was shown to offer some relief for HS symptoms in previous studies [6,12,18]. However, reported symptom relief ranged from 2% to >50.0% [12,25]. The lack of effectiveness of light-blocking masks in this study suggests that the actual trigger may not be daylight intensity, but increased daylight hours, which is supported by the seasonal initiation of symptoms coincident with spring and summer months reported here and elsewhere [1,2]. Photoperiod signals are transmitted via neuroendocrine pathways that regulate melatonin production and in turn, GnRH release [43,44]. Geldings lack the ability to regulate the increased release of GnRH due to the absence of negative feedback via testosterone production by the testes. A study evaluating administration of a GnRH vaccine to treat HS symptoms in geldings showed no effect of treatment, but the authors suggested that results were confounded by low study numbers and poor owner compliance [30]. Similarly, attempts to manipulate photoperiod signals through administration of melatonin were reported as successful in only two out of seven cases [6]. Evening doses of melatonin were begun in the spring and ceased in the autumn to mimic a short daylength and block the seasonal rise in GnRH [6]. These previous attempts to manipulate seasonal signals were aimed at maintaining a short-day signal [6,30]. Providing continuous extended day length may represent a more effective alternative means of manipulating the seasonal signal for treatment of HS and merits further investigation. This is tentatively supported by a report of complete resolution of HS symptoms by a single respondent who used a mobile device to deliver a long-day photoperiod signal between autumn and spring. 

Bodywork is a general term used to describe a wide range of holistic treatments, such as acupuncture, craniosacral therapy, physiotherapy and chiropractic adjustment. Madigan and Bell [4] reported that of 26% of horses treated by a chiropractor, only 1 responded to the treatment. Low reports of efficacy were also found in the current study, with the exception of one respondent who reported resolution of headshaking symptoms in response to craniosacral therapy. Kanik et al. (2017) [45] hypothesised that craniosacral therapy may be a treatment option for HS, but research evidence is lacking.

Pharmaceutical compounds were used by 40.3% of respondents but none were considered effective as a sole treatment for HS in this study. Cyproheptadine has been used to treat HS neuropathic pain [4,6,9,46] but was generally ineffective unless combined with carbamazepine and/or when administered at higher doses [47].

Only one survey respondent reported use of an unspecified surgical treatment, in addition to other treatments for HS, such that its effectiveness could not be determined. Surgical treatments for HS have been proposed where trigeminal neuralgia is suspected [9,21,48]. However, low use of these treatment options has also been reported in other surveys [1,18,27] presumably because of the high costs involved and limited published evidence of success [21,27].

The finding that so many Australian owners used more than one treatment option strongly suggests the failure to successfully mitigate the symptoms of HS with any single treatment. The currently available treatment options in Australia and the perceived lack of efficacy demonstrate the importance of continuing to investigate alternative therapies that can be evaluated independently and quantitatively for efficacy. 

Differences in environment, housing, management conditions, the diversity of triggering factors for the condition and the inherent reliability issues related to owner reporting and symptom assessment continue to pose challenges for research. Single treatment studies in controlled environments using horses with a veterinary diagnosis of TMHS (following elimination of all other causalities using suitable diagnostic testing) represent the gold standard but remain practically challenging. 

## 5. Conclusions

This study provides the first overview of headshaking syndrome (HS) symptomatology, seasonality and triggers, as well as treatments and their perceived efficacy in the Australian horse population. The presented results further contribute to our understanding of the condition. In keeping with the results of HS horse owner surveys in the Northern Hemisphere, geldings were more often affected compared to mares, seasonal appearance of symptoms was common, and primary environmental triggers were in agreement. A novel finding of the current study was the later symptom onset in geldings, which could relate to the age at which males were gelded. While bright sunlight was the most commonly reported trigger by Australian owners, light-blocking masks were generally considered ineffective suggesting that day length, rather than light intensity, may influence light-related headshaking behaviours. Most respondents reported using a combination of treatments with varying degrees of efficacy. Importantly, a third of respondents found all treatments to be ineffective, demonstrating a clear need for further research into alternative treatments for this condition that significantly impacts horse welfare and horse–owner relationships.

## Figures and Tables

**Table 1:** Sex distribution, mean age, age of onset and number of years horses have been exhibiting headshaking syndrome symptoms. Non-parametric Mann–Whitney tests were conducted to assess differences between geldings and mares for age at time of survey, age at symptom onset and years with HS symptoms, and resultant *p*-values are presented.

	Sex		Age at Time of Survey			Age at Symptom Onset			Years with HS Symptoms		
	N	Percentage (%)	n/N	Mean (±SD)	*p*-Value	n/N	Mean (±SD)	*p*-Value	n/N	Mean (±SD)	*p*-Value
**Total**	216	100	214/216	13.1 (±5.8)	N/A	201/216	9.6 (±4.7)	N/A	198/216	3.6 (±4.1)	N/A
**Mares**	51	23.6	51/214	11.3 (±5.7)	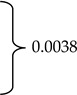	44/201	7.9 (±4.0)	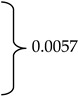	43/198	3.2 (±4.9)	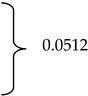
**Geldings**	164	75.9	162/214	13.7 (±5.7)		156/201	10.0 (±4.7)		154/198	3.7 (±3.8)	
**Stallion**	1	<1.0	1/214	24 *	N/A	1/201	20 *	N/A	1/198	4 *	N/A

* The stallion (n = 1) was not included in the non-parametric Mann–Whitney tests. Age, age at symptom onset and number of years of HS symptoms are reported. HS = headshaking syndrome, n = number of respondents, N = total number of survey respondents, SD = standard deviation, N/A = not applicable.

**Table 2:** Prevalence of specific headshaking syndrome symptoms among the study population.

Headshaking Syndrome Symptoms		
Symptom	Number of Horses	Percentage (%)
Tossing head up and down	172	79.6
Rubbing face on legs	143	66.2
Vertical ticking (flicking head towards chest)	128	59.2
Snorting regularly and/or sneezing	102	47.2
Rubbing face on objects	101	46.8
Anxiety	71	32.8
Tightness of muzzle/mouth/lower lip	70	32.4
Striking at head with front feet	56	25.9
Pushing face into trees/bushes or similar	52	24.1
Tossing head in circles	31	14.4
Depression	25	11.6
Other	43	19.9

## Data Availability

Collated data related to anonymized survey responses are available upon request.

## Supplementary Materials

The survey can be downloaded at: https://github.com/EquineResearch/Headshaking-Syndrome-Australian-Survey.
